# Genome-wide identification of long non-coding RNAs and their potential functions in radish response to salt stress

**DOI:** 10.3389/fgene.2023.1232363

**Published:** 2023-11-02

**Authors:** Xiaochuan Sun, Mingjia Tang, Liang Xu, Xiaobo Luo, Yutong Shang, Weike Duan, Zhinan Huang, Cong Jin, Guodong Chen

**Affiliations:** ^1^ School of Life Science and Food Engineering, Huaiyin Institute of Technology, Huaian, China; ^2^ Department of Horticulture, Zhejiang University, Hangzhou, China; ^3^ National Key Laboratory of Crop Genetics & Germplasm Enhancement and Utilization, Key Laboratory of Horticultural Crop Biology and Genetic Improvement (East China) of MOAR, College of Horticulture, Nanjing Agricultural University, Nanjing, China; ^4^ Guizhou Institute of Biotechnology, Guizhou Province Academy of Agricultural Sciences, Guiyang, China

**Keywords:** lncRNA, salt stress, *Raphanus sativus* L., RNA-seq, target genes, Gene Ontology and pathway analyses

## Abstract

Long non-coding RNAs (lncRNAs) are increasingly recognized as cis- and trans-acting regulators of protein-coding genes in plants, particularly in response to abiotic stressors. Among these stressors, high soil salinity poses a significant challenge to crop productivity. Radish (*Raphanus sativus* L.) is a prominent root vegetable crop that exhibits moderate susceptibility to salt stress, particularly during the seedling stage. Nevertheless, the precise regulatory mechanisms through which lncRNAs contribute to salt response in radish remain largely unexplored. In this study, we performed genome-wide identification of lncRNAs using strand-specific RNA sequencing on radish fleshy root samples subjected to varying time points of salinity treatment. A total of 7,709 novel lncRNAs were identified, with 363 of them displaying significant differential expression in response to salt application. Furthermore, through target gene prediction, 5,006 cis- and 5,983 trans-target genes were obtained for the differentially expressed lncRNAs. The predicted target genes of these salt-responsive lncRNAs exhibited strong associations with various plant defense mechanisms, including signal perception and transduction, transcription regulation, ion homeostasis, osmoregulation, reactive oxygen species scavenging, photosynthesis, phytohormone regulation, and kinase activity. Notably, this study represents the first comprehensive genome-wide analysis of salt-responsive lncRNAs in radish, to the best of our knowledge. These findings provide a basis for future functional analysis of lncRNAs implicated in the defense response of radish against high salinity, which will aid in further understanding the regulatory mechanisms underlying radish response to salt stress.

## Introduction

Soil salinity is one of the major damaging issues to cultivable land, resulting in an inevitable slowdown to plant growth and yields (Safdar et al., 2019). It is estimated that approximately 20% of global irrigated land is affected by salinization, which negatively affects land productivity (Islam et al., 2022). Hyper-ionic and hyper-osmotic injuries, as well as oxidative damage caused by prolonged saline conditions, are the main effects of salt stress in plants. To a certain degree, plants have evolved adaptive mechanisms to ensure their survival under high salinity through molecular, biochemical, and physiological adjustments. Hence, it is imperative to unravel the molecular and physiological mechanisms associated with salt stress tolerance in order to address the substantial agronomic challenges posed by salinization.

Long non-coding RNAs (lncRNAs) refer to a type of non-coding RNAs (ncRNAs) greater than 200 nucleotides (nt) in length without discernible coding potential in eukaryotes (Kapranov et al., 2007; Wang and Chekanova, 2017). Similar to classical messenger RNAs (mRNAs), lncRNAs are primarily transcribed by RNA Pol II, capped, polyadenylated, and usually spliced, but generally exhibit lower expression levels (Chekanova, 2015; Quan et al., 2015). In plants, a subset of lncRNAs is also transcribed by two other plant-specific RNA polymerases, namely, Pol IV and V, and is usually synthesized from transposable elements (St Laurent et al., 2015; Wierzbicki et al., 2021). Based on their genomic location and cellular function, lncRNAs can be classified into sense and antisense lncRNAs, intronic lncRNAs, and intergenic lncRNAs (lincRNAs) (Ma et al., 2013). Substantial evidence supports the notion that lncRNAs play crucial roles as regulators in various biological processes, exerting their influence on gene expression in both cis- and trans-acting manners (Gil and Ulitsky, 2020; Jha et al., 2020); they can serve as molecular signals, decoys, guides, and scaffolds (Wang and Chang, 2011).

Initially, lncRNAs were neglected as a component of transcriptional noise (Chen et al., 2018); nevertheless, accumulating evidence has manifested that lncRNAs play crucial roles in multiple plant biological events, including photomorphogenesis (Wang et al., 2014; Sun et al., 2020), senescence (Huang et al., 2021), reproduction (Zhang et al., 2014; Zhou et al., 2022a), seed aging (Zhang et al., 2022), and fruit ripening (Tian et al., 2019; Zhou et al., 2022b). To date, there have been experimental investigations into the functions of specific plant lncRNAs. For instance, lncRNA39026 in tomato may possess the capability to impact decoy miR168a, leading to an increase in the expression of pathogenesis-related genes, thereby improving disease resistance (Hou et al., 2020). In rice, an intronic lncRNA known as RICE FLOWERING ASSOCIATED (RIFLA) plays an essential role in the flowering process mediated by *OsMADS56*, achieved through the formation of a complex with *OsiEZ1* (Shin et al., 2022). Recent research has hinted that lncRNAs exhibit responsiveness to various stressors, including salt stress (Baruah et al., 2021; Cao et al., 2021; Liu et al., 2022a; Hu et al., 2022). For example, a total of 742 salt-responsive lncRNAs were identified in maize (Liu et al., 2022a). Similarly, in *Spirodela polyrhiza*, a total of 2,815 lncRNAs were discovered under salt stress conditions, out of which 185 exhibited differential expression in response to salinity (Fu et al., 2020). In the context of cotton, the functional role of lncRNA973 in fine-tuning salt stress response has been experimentally verified (Zhang et al., 2019). Additionally, the overexpression of lncRNA77580 in *Glycine max* has been found to regulate the response to both salt and drought stress by modulating the transcription of distinct sets of stress-related genes (Chen et al., 2023). Overall, these reports provide substantial evidence to affirm the significant role of lncRNAs in the regulation of plant stress tolerance.

Radish (*Raphanus sativus* L.), an essential root vegetable belonging to the Brassicaceae family, is cultivated globally owing to its high nutritional and medicinal values. Among vegetable crops, radish exhibits moderate sensitivity to salt, with its edible fleshy taproots displaying notable responsiveness to salt stimulus up to a maximum soil salinity threshold of 1.2 dS/m (Grattan et al., 2016). The yield and quality of radish taproots are significantly impacted by salt stress, as it has become the major limiting factor due to soil salinization and secondary salinization. Investigating the response of radish to salt stress holds potential for the advancement of salt-tolerant radish lines in the field of radish breeding. In radish, a substantial number of miRNAs and protein-coding genes implicated in salt adaptation have been reported in our previous studies (Sun et al., 2015; 2016). Nevertheless, the reports on lncRNAs involved in salt stress response remain elusive in radish. Fortunately, with the notable advancements in deep transcriptome sequencing technology and associated bioinformatics methodologies, it is now easier to comprehensively mine novel non-coding RNA molecules. In this study, strand-specific RNA sequencing (ssRNA-seq) was utilized to identify and characterize the salt-responsive lncRNAs in radish. Furthermore, the function of differentially expressed lncRNAs (DE-lncRNAs) was investigated by examining their position or co-expression associations with target genes. These findings could serve as a starting point for discerning the role of lncRNAs in the regulatory mechanisms governing salt stress response in radish, thereby offering potential benefits for the development of salt-tolerant radish cultivars.

## Materials and methods

### Plant materials and salt treatments

The radish advanced inbred line, ‘YH’, was used in this study. The seeds were rinsed and surface-sterilized using a 1.2% NaClO solution before germinating on moist filter paper and further incubated at 25°C in darkness for 2 days. Subsequently, the seedlings were transferred to plastic pots and grown at 25°C/18°C with a relative humidity of 60% in a 14 h light/10 h dark cycle. Uniform four-leaf-old seedlings were transferred into hydroponic conditions with half-strength Hoagland’s solution. After acclimating for a 1-week slow seeding period, the plants were stressed with 200 mM NaCl. The materials for transcriptome sequencing were collected at 0 h (control), 6 h, 12 h, 24 h, 48 h, and 96 h under salt treatment with two biological replicates at each time point, and the sample for each replicate contained an equal amount of fleshy taproot from three individual seedlings. Additionally, the samples were prepared for qualitative real-time polymerase chain reaction (qRT-PCR) analysis in triplicate at every time point during salt treatments, with three individual seedlings per replicate. Last, the samples were frozen in liquid nitrogen immediately and stored at −80°C until use.

### RNA extraction, quality control, and cDNA library preparation

The construction of transcriptome libraries and deep sequencing were implemented at the Novogene Bioinformatics Institute (Beijing, China). The isolation of total RNAs was performed following the manufacturer’s recommended protocol (Invitrogen, Carlsbad, CA, United States). RNA quality and integrity were monitored using the Agilent 2100 Bioanalyzer. Ribosomal RNA was filtered using the Ribo-Zero™ rRNA Removal Kit (Epicentre, United States). The cDNA libraries were generated using the NEBNext^®^ Ultra^™^ II RNA Kits (NEB, United States).

### Sequencing and bioinformatic discovery of lncRNAs

The constructed libraries underwent sequencing using the Illumina NovaSeq PE150 platform. To obtain clean reads, the raw reads were filtered with fastp to eliminate adapter sequences and reads containing poly-N and low-quality reads. Meanwhile, the Q20, Q30, and GC contents were calculated. The clean reads were then aligned to the radish reference genome Rs1.0 assembly (http://radish-genome.org/) using HISAT2 (version 2.1.0) (Kim et al., 2015). Based on the assembled transcripts, the candidate lncRNAs were identified using a rigorous set of criteria. Initially, transcripts possessing a class_code of “i”, “u”, “x”, and “o” were reserved. Subsequently, transcripts exceeding a length of 200 bp and containing a minimum of two exons were retained. Finally, transcripts exhibiting overlap with established protein-coding domains were removed, while those overlapping with annotated lncRNAs were preserved. The coding potential of the retained transcripts was assessed using the Coding Potential Calculator (CPC2), Coding-Non-Coding-Index (CNCI), and Pfam. Non-coding transcripts shared by these three predictive software packages were identified as novel lncRNAs.

### Differential expression analysis

The StringTie program (version 1.3.4) using the “-G option” (Pertea et al., 2015), in conjunction with the calculated FPKM values, was performed to estimate the expression level of both lncRNAs and coding genes in each sample. The DESeq2 R package (version 1.10.1) (Love et al., 2014) was applied to identify differentially expressed genes (DEGs) and DE-lncRNAs between the control and salinity treatments. Genes or lncRNAs with adjusted *p*-values (p_adj_) ≤ 0.05 and log_2_ (|fold change|) ≥ 1 were determined as DEGs and DE-lncRNAs, respectively.

### Potential target gene prediction and enrichment analysis of DE-lncRNAs

In order to enhance the understanding of lncRNA functions in radish, the potential cis- and trans-target mRNAs of DE-lncRNAs were predicted. According to the genomic location of lncRNAs relative to the neighboring genes, the target mRNAs in the 100-kb region upstream or downstream of DE-lncRNAs were regarded as potential cis-target genes. Pearson’s correlation coefficient (PCC) between DE-lncRNAs and the corresponding transcripts was calculated based on their co-expression to predict the trans-target genes of lncRNAs. The transcripts up to the strict standards of (|PCC| > 0.8, *p*-value < 0.01) were defined as the trans-target genes of DE-lncRNAs. Subsequently, Gene Ontology (GO) and Kyoto Encyclopedia of Genes and Genomes (KEGG) pathway analyses were carried out to further annotate the functions of cis- and trans-target genes for DE-lncRNAs. The GO terms and KEGG pathways with corrected *p*-values ≤ 0.05 were recognized as significantly enriched.

### Validation of DE-lncRNAs by qRT-PCR analysis

In order to validate the findings from RNA-seq analysis, a total of six DE-lncRNAs, namely, TCONS_00053136, TCONS_00062931, TCONS_00081369, TCONS_00101865, TCONS_00122106, and TCONS_00159796, were randomly selected for qRT-PCR analysis based on their significant differential expression observed in the RNA-seq data under salt stress conditions. The specific primers used for qRT-PCR analysis are listed in Supplementary Table S1. The expression of the DE-lncRNAs was normalized using *RsActin* as the internal reference. qRT-PCRs were operated on a LightCycler R 480 System (Roche, Mannheim, and Germany) using the 2^−ΔΔ*C*_T_^ method.

## Results

### Overview of whole-transcriptome sequencing in radish

To comprehensively identify the lncRNAs responsive to salt stress in radish, high-throughput ssRNA-seq of twelve libraries derived from samples of both the control (CK) and salinity treatment groups (NA_1-NA_5) were analyzed. The raw sequencing data have been deposited in the Sequence Read Archive (SRA) at the NCBI, with BioProject No. PRJNA930138. Following quality control measures on the raw data, a total of 1.11 billion clean reads (with an average of 92.71 million reads) were obtained. The Q20 and Q30 scores exceeded 90%, and the GC content ranged from 45.31% to 48.98%. All clean reads were aligned to the radish reference genome, with the alignment ratio ranging from 70.35% to 79.85%. The statistics of sequencing and mapping for each library are summarized in Supplementary Table S2.

### Identification of novel lncRNAs in radish

**FIGURE 1:**
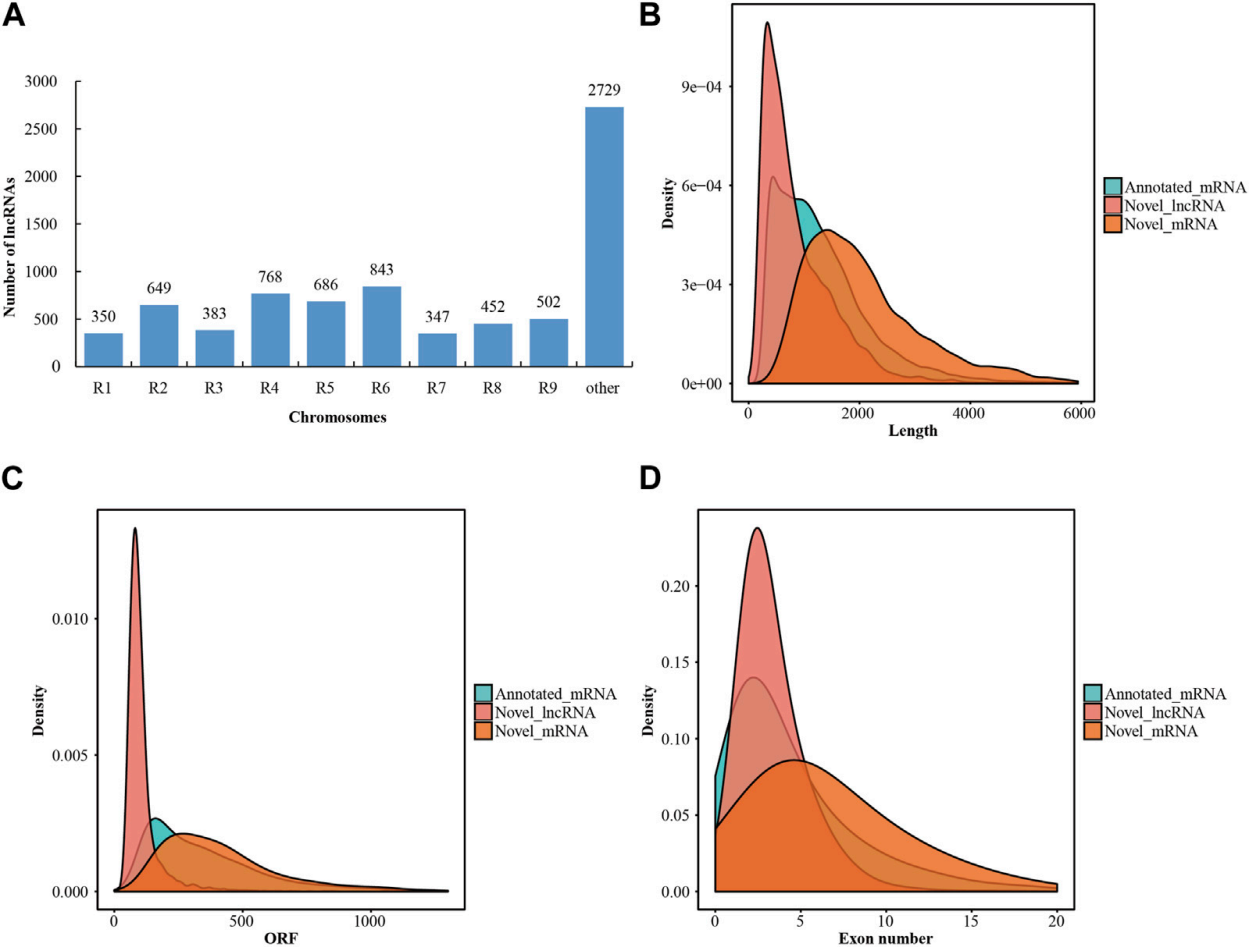
Characteristics of lncRNAs in radish. **(A)** Distribution of lncRNAs on different chromosomes. **(B)** Statistical results of length distribution between the lncRNAs and mRNAs. **(C)** Statistical results of ORF distribution between lncRNAs and mRNAs. **(D)** Statistical results of exon number percentages between lncRNAs and mRNAs.

After transcriptome assembly using the StringTie software, a total of 708,647 assembled transcripts were obtained from the mapped reads of the twelve libraries. To predict the coding potential of these novel assembled transcripts, two prediction software programs (CPC2 and CNCI) and one database (Pfam) were employed. Consequently, 7,709 transcripts were identified as novel lncRNAs (Supplementary Table S3). Meanwhile, 8,288 transcripts were determined to be novel mRNAs (Supplementary Table S4). The lncRNAs were characterized based on their relative proximity to the nearest protein-coding gene neighbor, with all lncRNAs located as lincRNAs in the intergenic region. Approximately two-thirds of the lncRNAs were distributed across all the radish chromosomes, leaving more than one-third of the lncRNAs unmapped to any specific chromosome (Figure 1A; Supplementary Table S3). In addition, a comparative analysis was conducted to assess the fundamental characteristics of lncRNAs and mRNAs, encompassing sequence length, open reading frames (ORFs), and exon numbers. The length distribution of lncRNAs in radish predominantly fell within the range of 200–7,000 bp, with approximately 93.4% of lncRNAs falling within the 200–2,000 bp range (Figure 1B). Additionally, radish lncRNAs also harbored shorter ORFs compared to mRNAs, with the majority of novel lncRNAs having ORFs of less than 150 nucleotides (Figure 1C). Structural analysis further indicated that a significant proportion (67.4%) of novel lncRNAs possessed fewer than three exons, whereas mRNAs displayed a higher exon count (Figure 1D).

### Identification and analysis of DE-lncRNAs in radish

**FIGURE 2:**
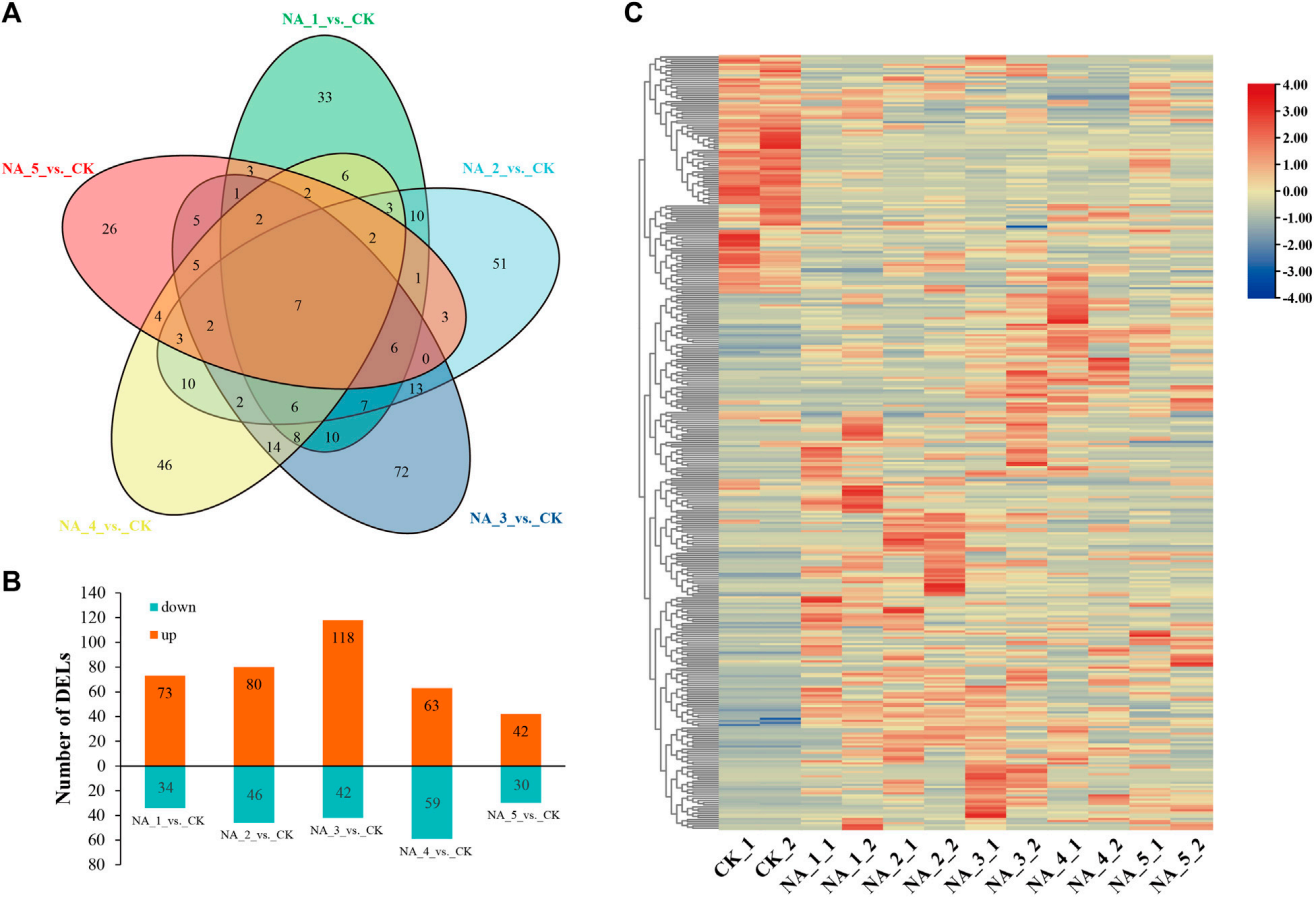
Overall view of DE-lncRNAs under different salt conditions in radish. **(A)** Venn diagram of DE-lncRNAs. **(B)** Number of upregulated/downregulated lncRNAs. **(C)** Hierarchical clustering plot of all DE-lncRNAs based on the average FPKM value of each set of replicates.

In order to investigate the changes in transcription of lncRNAs during salt stress, we conducted a comprehensive analysis using the edgeR package (version 3.12.1). DE-lncRNAs were filtered based on the conditions p_adj_ ≤ 0.05 and log_2_(|fold change|) ≥ 1. In total, 363 DE-lncRNAs were identified, with 107, 126, 160, 122, and 72 members detected in the NA_1_vs._CK, NA_2_vs._CK, NA_3_vs._CK, NA_4_vs._CK, and NA_5_vs._CK comparisons, respectively (Supplementary Table S5). Only seven common DE-lncRNAs were traced in five comparisons (Figure 2A). In the comparison between NA_1_vs._CK, a total of 73 lncRNAs were found to be upregulated, while 34 lncRNAs were downregulated. Similarly, in the comparison between NA_2_vs._CK, 80 lncRNAs were identified as upregulated and 46 lncRNAs were downregulated. Furthermore, in the comparison between NA_3_vs._CK, a total of 118 lncRNAs were upregulated and 42 lncRNAs were downregulated. Additionally, in the comparison between NA_4_vs._CK, 63 lncRNAs were discovered to be upregulated and 59 lncRNAs were downregulated. Last, in the comparison between NA_5_vs._CK, 42 lncRNAs were found to be upregulated and 30 lncRNAs were downregulated. Consequently, the prevalence of upregulated lncRNAs was observed in all comparisons, as depicted in Figure 2B. Furthermore, a heat map was employed to aggregate the DE-lncRNAs, enabling the visualization of expression patterns across all six time points of salt treatments (Figure 2C).

### Analysis of potential target genes of DE-lncRNAs in radish

To explore the potential biological roles of lncRNAs in response to salinity stressors in radish, the target genes fine-tuned by DE-lncRNAs were identified using both cis- and trans-acting approaches (Supplementary Table S6). In terms of cis-regulation, a total of 5,006 target genes were observed for 303 DE-lncRNAs depending on their genomic location, resulting in 6,774 lncRNA–mRNA interaction pairs. Additionally, 5,983 trans-target genes were recognized for 249 DE-lncRNAs according to their co-expression relationships, resulting in 24,059 lncRNA–mRNA interaction pairs. Notably, 599 target genes were found to be jointly regulated by DE-lncRNAs via both cis- and trans-acting approaches. Moreover, the study found that 89 target genes (1.78%) exhibited differential expression under salt stress with 121 cis-regulatory pairs, and 229 target genes (3.83%) showed differential expression with 1,712 trans-regulatory pairs (Tables 1, 2; Supplementary Table S7). To further distinguish the potential key target genes responsive to salt stress, further annotation and analysis were conducted on these 121 cis-regulatory pairs and 1,712 trans-regulatory pairs. Among the differentially expressed targeted genes, there were crucial genes encoding for transcription factors (TFs) (e.g., bHLH, WRKY, MYB, and MADS-box), transport-related proteins (e.g., calcium-transporting ATPase, vacuolar glucose transporter, annexin, sugar transporter, and sulfate transporter), cytochrome P450, receptor-like protein kinases, enzymes associated with signal transduction (e.g., mitogen-activated protein kinase, calmodulin, CBL-interacting protein kinase, and CBL-interacting serine/threonine-protein kinase), enzymes related to photosynthesis (e.g., water-soluble chlorophyll protein), plant hormone regulation (e.g., ACC oxidase, auxin-responsive GH3 family protein, and proline dehydrogenase), osmoregulatory factors like trehalose-6-phosphate phosphatase, and enzymes implicated in mitigating oxidative damage (e.g., superoxide dismutase, peroxidase, and thioredoxin). All in all, these findings hinted that the identified DE-lncRNA-target pairs may play essential roles in regulating the significant biological processes related to the response of radish to salt stress.

**TABLE 1:** Representative differentially expressed cis-targets for some salt-responsive lncRNAs.

lncRNA_ID	Gene_ID	Description of the target gene	Gene name	Distance	cis location
TCONS_00007404	Rs013430	Receptor-like protein kinase-like	—	14,586	Upstream
TCONS_00010534	Rs038050	TSK-associating protein 1	*TSA1*	13,490	Downstream
TCONS_00015424	Rs068580	Leucine-rich repeat (LRR) family protein	—	75,018	Downstream
TCONS_00020107	Rs069760	Protein TIFY 11b	—	87,973	Upstream
TCONS_00021725	Rs228720	LRR family protein FLR1	*FLR1*	94,115	Downstream
TCONS_00034882	XLOC_021821	Ubiquitin-related modifier 1 homolog 1	—	10,293	Downstream
TCONS_00035170	XLOC_018600	Mitogen-activated protein kinase 20-like	*MAPK20*	−32,989	Antisense
TCONS_00036718	Rs133050	Calcium-transporting ATPase 8	—	9,985	Downstream
TCONS_00049496	Rs159740	ACT domain repeat 7	*ACR7*	37,198	Downstream
	Rs159870	Trehalose-6-phosphate phosphatase	*TPPB*	41,249	Upstream
TCONS_00053221	XLOC_033420	Calmodulin-1	*CAM1*	−2,821	Sense
TCONS_00053239	XLOC_033420	Calmodulin-1	*CAM1*	82,458	Upstream
TCONS_00058325	Rs223580	Protein dehydration-induced 19-4	—	17,702	Upstream
TCONS_00060741	Rs240270	F-box kelch-repeat protein	—	74,856	Downstream
TCONS_00066609	XLOC_047803	Cytochrome P450 72A15-like	—	−2,858	Antisense
	XLOC_047805	Cytochrome P450 72A15-like	—	3,692	Downstream
TCONS_00069122	XLOC_037292	Cytochrome P450 72A13	—	33,818	Downstream
TCONS_00069410	Rs232150	JA-responsive protein 1	*JAC1*	7,495	Downstream
TCONS_00069424	Rs232150	JA-responsive protein 1	*JAC1*	70,884	Upstream
TCONS_00071696	XLOC_038932	Calcium-transporting ATPase 3, endoplasmic reticulum-type-like	—	−13,900	Antisense
TCONS_00079480	Rs306040	F-box family protein	*FBS1*	84,994	Upstream
TCONS_00096570	XLOC_053988	Receptor-like protein 12	*RLP12*	37,622	Downstream
TCONS_00110905	XLOC_073149	Probable LRR receptor-like serine/threonine-protein kinase	—	43,945	Downstream
TCONS_00123910	Rs478440	Auxin-responsive GH3 family protein	—	4,097	Downstream
TCONS_00126664	XLOC_079470	Transcription factor bHLH96-like	*bHLH96*	5,833	Downstream
	XLOC_083827	Autophagy-related protein 18h-like	—	41,217	Upstream
TCONS_00127122	XLOC_084067	Ethylene-responsive transcription factor ABR1-like	—	68,396	Upstream
TCONS_00138360	Rs510300	CBL-interacting protein kinase 25	*CIPK25*	3,660	Upstream
	XLOC_087207	Cytochrome P450 71B11	—	−4,846	Sense
TCONS_00149969	XLOC_095877	Enhanced disease resistance 2-like	—	513	Upstream

**TABLE 2:** Representative differentially expressed trans-targets for some salt-responsive lncRNAs.

lncRNA_ID	mRNA_gene_ID	Description of the target gene	Gene name
TCONS_00007404	Rs069590	Lipoxygenase 3	*LOX3*
	Rs145870	Polygalacturonase inhibiting protein 1	*PGIP1*
	Rs159870	Trehalose-6-phosphate phosphatase	*TPPB*
	Rs232150	JA-responsive protein 1	*JAC1*
	Rs269180	TSK-associating protein 1	*TSA1*
	Rs375400	WRKY transcription factor 40	*WRKY40*
	Rs450390	Nitrate reductase 1	*NIA1*
	XLOC_072566	ABC transporter G family member 35	—
TCONS_00021574	Rs045300	Aminopeptidase M1	*APM1*
	Rs388430	Transcription factor MYB75	*MYB75*
TCONS_00024017	XLOC_021106	E3 ubiquitin-protein ligase RHA1B-like	—
TCONS_00031201	Rs038050	TSK-associating protein 1	*TSA1*
	Rs086530	Trehalose-6-phosphate phosphatase	*TPPB*
	Rs119710	Peroxidase	*POD*
	Rs228720	Leucine-rich repeat protein FLR1	*FLR1*
	XLOC_004367	ABC transporter G family member 35	—
TCONS_00036276	Rs251480	Water-soluble chlorophyll protein	*WSCP1*
	XLOC_076218	Abietadienol/abietadienal oxidase	—
TCONS_00036702	Rs142820	Protein TIFY 9	—
	Rs305010	Cytochrome P450	—
	Rs399730	Glutamate decarboxylase 1	*GAD1*
	XLOC_095772	Receptor-like protein kinase 2	*RPK2*
TCONS_00038561	Rs009970	ACC oxidase	—
	Rs290510	Vacuolar glucose transporter 1	*VGT1*
	Rs391910	Polygalacturonase inhibiting protein 1	*PGIP1*
	Rs424800	Transcription factor bHLH35	*bHLH35*
TCONS_00039084	XLOC_065872	Chalcone synthase 3	*CHS3*
TCONS_00043052	XLOC_001705	Zinc finger protein DOF1.3	*DOF1.3*
TCONS_00044336	XLOC_102899	Putative expansin-B2	*EXPB2*
TCONS_00047111	Rs096180	Pectate lyase	—
TCONS_00052720	Rs057150	Superoxide dismutase	*SOD*
TCONS_00056164	Rs155060	Xyloglucan endotransglucosylase/hydrolase 18	*XTH18*
	Rs306040	F-box family protein	*FBS1*
TCONS_00058325	XLOC_100431	Endoglucanase 19	—
TCONS_00060741	Rs236670	Transducin/WD40 repeat-like superfamily protein	—
	Rs478440	Auxin-responsive GH3 family protein	—
	Rs492760	Allene oxide cyclase 2	*AOC2*
TCONS_00071951	Rs392590	CBL-interacting protein kinase 2	*CIPK2*
	Rs412870	Lipoxygenase 3	*LOX3*
	Rs450300	Trehalose-6-phosphate phosphatase	*TPPB*
TCONS_00073919	Rs357900	Chitinase family protein	—
TCONS_00073924	XLOC_024931	MADS-box protein AGL24-like	—
TCONS_00081104	Rs211640	Cytochrome b5	—
TCONS_00094358	Rs074390	Pyruvate decarboxylase	*PDC1*
	Rs194210	bHLH transcription factor	—
TCONS_00100832	Rs492750	Allene oxide cyclase 3	*AOC3*
TCONS_00101865	XLOC_028048	Phytosulfokines 2	*PSK2_1*
	XLOC_041486	Calmodulin-like protein 11	*CML11*
TCONS_00115773	Rs119330	Annexin d4	*ANN4*
	Rs315450	Proline dehydrogenase	—
TCONS_00123532	XLOC_086136	Probable mannan synthase 7	—
TCONS_00133489	Rs069810	Lipoxygenase 3	*LOX3*
	Rs152410	Dehydration-responsive element-binding protein 3-like	—
	XLOC_027166	Squamosa promoter-binding-like protein 2	*SPL2*
TCONS_00137062	Rs006490	Sugar transporter EDR6-like 3	—
	Rs155070	Xyloglucan endotransglucosylase/hydrolase protein 20	*XTH20*
	Rs157300	Thioredoxin m4	—
TCONS_00138712	Rs132610	MATE efflux family protein	—
	Rs448590	WRKY transcription factor 40	*WRKY40*
TCONS_00140966	Rs043580	Mitogen-activated protein kinase kinase kinase 21	*MAPKKK21*
TCONS_00146159	Rs432680	Ethylene-responsive element-binding factor 1	*ERF1A*
TCONS_00147454	XLOC_012140	Chaperone protein dnaJ 8	—
TCONS_00152030	XLOC_075206	WD repeat domain-containing protein 83-like	—
TCONS_00152155	Rs501390	Proton-dependent oligopeptide transport family protein	—
TCONS_00155276	Rs269160	β-Glucosidase 18	*BGLU18*
TCONS_00159115	Rs067500	Sulfate transporter	—
	Rs159740	ACT domain repeat 7	*ACR7*
	Rs203420	Jacalin-related lectin 22	*JRL22*
	Rs458200	Phosphoinositide phospholipase C4	—
TCONS_00160055	Rs541640	Transducin/WD40 repeat-like superfamily protein	—
TCONS_00160242	XLOC_006145	CBL-interacting serine/threonine-protein kinase 17	*CIPK17*

### Enrichment analysis for cis-acting target genes of DE-lncRNAs

GO analysis exhibited that the cis-target genes of DE-lncRNAs were enriched in 2,961 GO terms, including 1,677 biological processes (BPs), 376 molecular functions (MFs), and 908 cellular components (CCs) (Supplementary Table S8). Increasingly, GO analysis of cis-acting targets responsive to salt stress was closely enriched in BP terms related to photosynthesis (e.g., GO: 0015979: photosynthesis and GO: 0015994: chlorophyll metabolic process), signal transduction (e.g., GO: 0009966: regulation of signal transduction; GO: 0023051: regulation of signaling; GO: 0009755: hormone-mediated signaling pathway; and GO: 0006465: signal peptide processing), and antioxidant regulation (e.g., GO: 0000302: response to reactive oxygen species and GO: 0006979: response to oxidative stress). In the MF category, specific GO terms included numerous ion transport activities (e.g., GO: 0005216: ion channel activity; GO: 0005262: calcium channel activity; GO: 0005272: sodium channel activity; and GO: 0015079: potassium ion transmembrane transporter activity). Regarding the CC category, GO terms associated with the photosynthetic components (e.g., GO: 0009507: chloroplast; GO: 0009521: photosystem; GO: 0009522: photosystem I; and GO: 0009523: photosystem II) and ion transport channels (e.g., GO: 0034702: ion channel complex; GO: 0034703: cation channel complex; and GO: 0034706: sodium channel complex) were enriched. Nevertheless, only one MF term “glutamate-cysteine ligase activity (GO: 0004357)” was significantly enriched in the NA_3_vs._CK comparison group for cis-acting targets.

**FIGURE 3:**
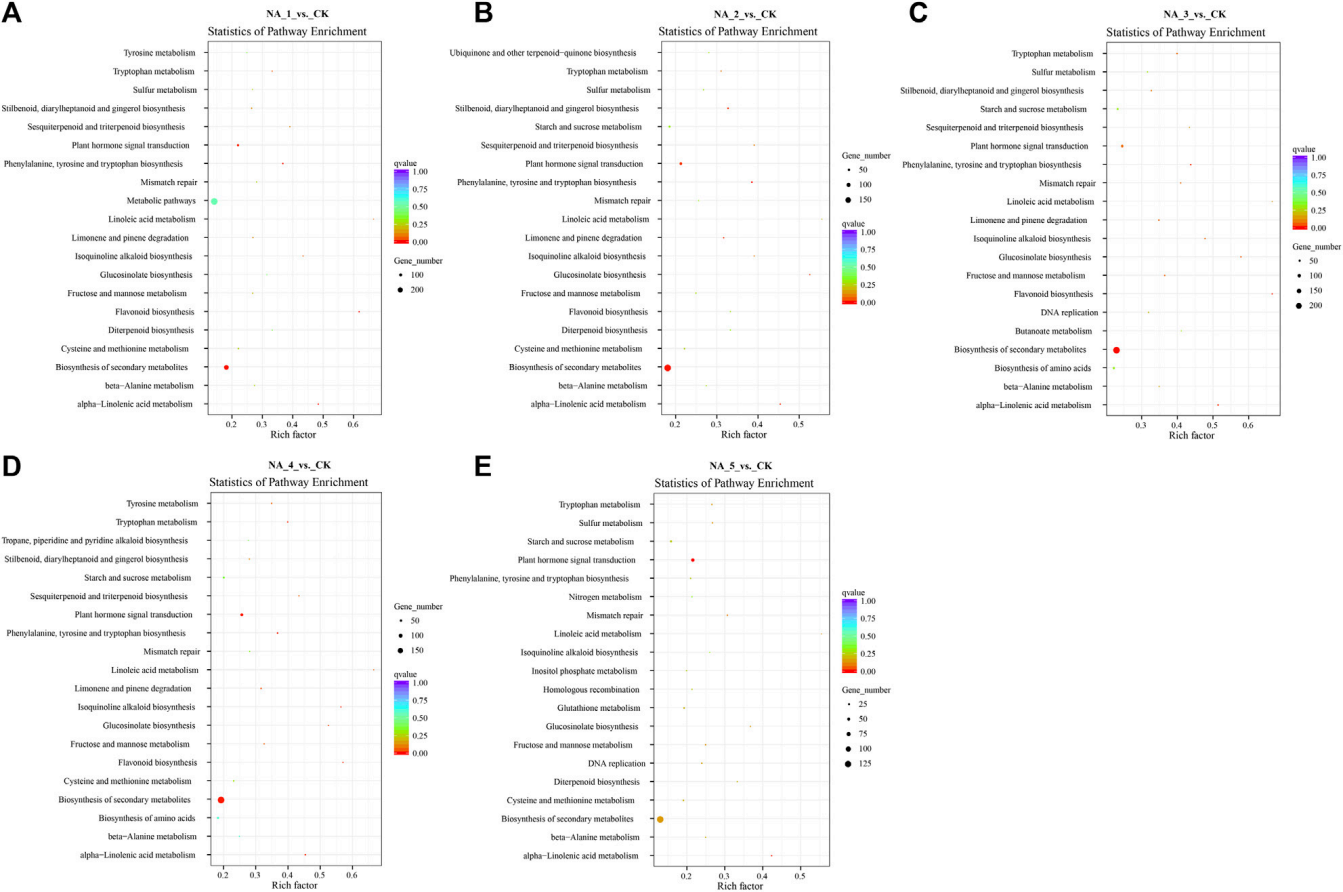
Top 20 enriched KEGG pathways for the cis-targets of DE-lncRNAs in the NA_1_vs._CK **(A)**, NA_2_vs._CK **(B)**, NA_3_vs._CK **(C)**, NA_4_vs._CK **(D)**, and NA_5_vs._CK **(E)** comparisons, respectively.

The analysis conducted using KEGG suggested that the cis-acting target genes of DE-lncRNAs were highly represented in 119 KEGG pathways (Figure 3; Supplementary Table S9). Especially, the top 20 enriched pathways in each comparison group demonstrated significant enrichment of key pathways such as plant hormone signal transduction, homologous recombination, ubiquitin-mediated proteolysis, and zeatin biosynthesis.

### Enrichment analysis for trans-acting target genes of DE-lncRNAs

Functional analysis suggested that the trans-acting target genes of DE-lncRNAs were enriched in 3,051 GO terms, including 1,710 BP terms, 418 MF terms, and 923 CC terms (Supplementary Table S10). As anticipated, the trans-targets of DE-lncRNAs exhibited a significant overlap in GO terms pertaining to essential biological processes (e.g., photosynthesis, signal transduction, and antioxidant regulation), molecular functions (e.g., ion transport activity), and cellular components (e.g., photosynthetic component and ion transport channel) with the cis-acting targets. Furthermore, a notable enrichment of 11, 14, 18, 15, and 7 GO terms was observed in the NA_1_vs._CK, NA_2_vs._CK, NA_3_vs._CK, NA_4_vs._CK, and NA_5_vs._CK comparisons, respectively (Supplementary Table S10). This demonstrates that the lncRNA-trans-regulatory target genes may play prior roles in response to salt stress in radish.

**FIGURE 4:**
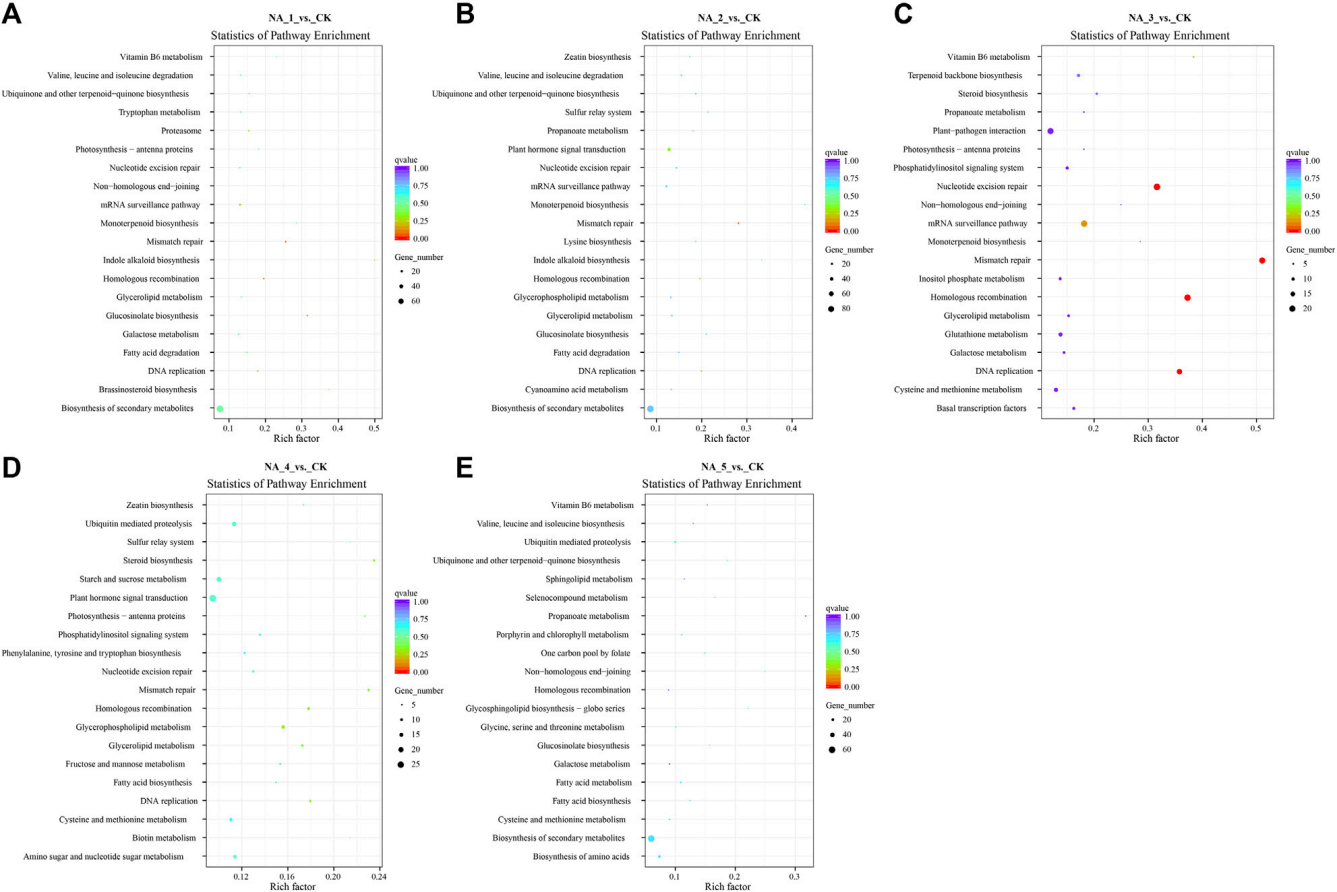
Top 20 enriched KEGG pathways for the trans-targets of DE-lncRNAs in the NA_1_vs._CK **(A)**, NA_2_vs._CK **(B)**, NA_3_vs._CK **(C)**, NA_4_vs._CK **(D)**, and NA_5_vs._CK **(E)** comparisons, respectively.

The trans-acting target genes of DE-lncRNAs exhibited enrichment in 118 KEGG pathways (the top 20 enriched pathways of each group are shown in Figure 4; Supplementary Table S11). Notably, several pathways, including α-linolenic acid metabolism, β-alanine metabolism, biosynthesis of secondary metabolites, fructose and mannose metabolism, glucosinolate biosynthesis, isoquinoline alkaloid biosynthesis, plant hormone signal transduction, and tryptophan metabolism, were significantly enriched across all comparison groups. Meanwhile, certain pathways associated with salt stress defense, such as plant hormone signal transduction, glutathione metabolism, ubiquinone, and other terpenoid-quinone biosynthesis, were also enriched for some target genes.

### Experimental validation of DE-lncRNAs

**FIGURE 5:**
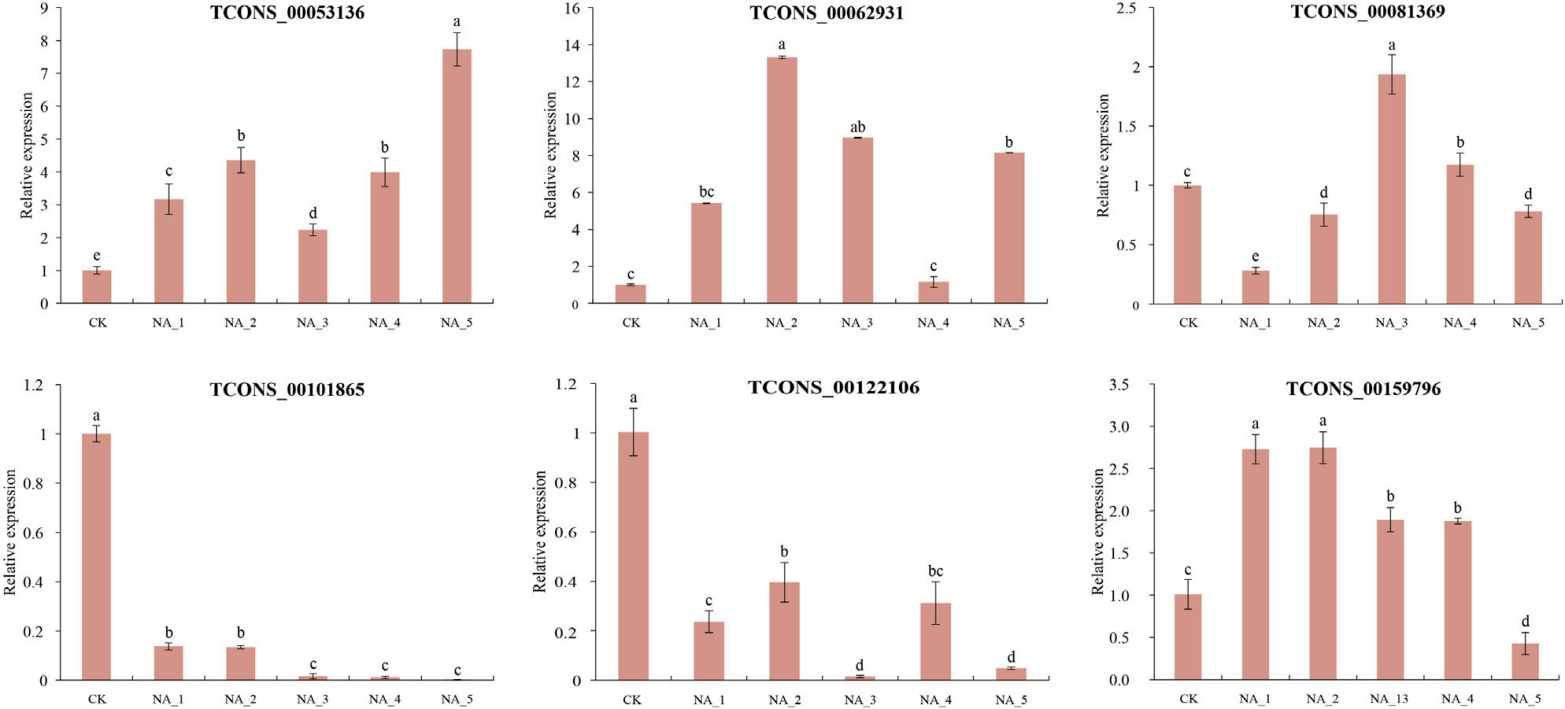
Expression validation of randomly selected DE-lncRNAs by the qRT-PCR assay. The significant difference was analyzed based on one-way ANOVA and Duncan’s multiple range tests with *p* < 0.05. Each bar shows the mean ± SE.

To validate the analysis of the data obtained from RNA-seq, six lncRNA transcripts were examined by qRT-PCR (Figure 5). In general, the expression patterns of four salt-responsive lncRNAs (TCONS_00053136, TCONS_00062931, TCONS_00101865, and TCONS_00122106) were relatively coincident with similar trends between qRT-PCR and RNA-seq, indicating the reliability of expression profiling based on RNA-seq data. However, the expression of TCONS_00081369 and TCONS_00159796 exhibited contrasting changes at certain time points during salt treatments when comparing the two methods. For instance, the upregulation of TCONS_00081369 at 12 h and 96 h of salt treatment was observed in the RNA-seq data, while a slight downregulation was detected in the qRT-PCR analysis. Conversely, TCONS_00159796 exhibited an opposite change in expression at 96 h under 200 mM NaCl. The observed disparity in expression levels between qRT-PCR and RNA-seq could be partially attributed to variations in sensitivity and algorithmic methodologies employed by these two approaches (Xu et al., 2015).

## Discussion

Salt stress is considered to be one of the main constraints on radish yield and quality. Recently, numerous investigations have highlighted the primary correlation between regulatory function and salt stress-responsive lncRNAs in plants (Zhang et al., 2019; Fu et al., 2020; Liu et al., 2022a; Chen et al., 2023). However, the potential role of lncRNAs during salt stress conditions in radish remains unexplored. In the current research, a total of 7,709 novel lncRNAs were systematically identified based on publicly accessible radish genome data using ssRNA-seq. Yet, in comparison to other plant species such as maize (Liu et al., 2022a), sesame (Gong et al., 2021), and peanut (Ma et al., 2020), only lincRNAs were observed in radish, manifesting that the lncRNA types may be highly specific to the context and species. Furthermore, it was also noticed that certain lncRNAs were not distributed on any chromosome, suggesting that these lncRNAs may be transcriptional noise (Nojima and Proudfoot, 2022). As anticipated, lncRNAs identified in radish were found to be shorter in total length and ORFs, and they also harbored fewer exons compared to protein-coding transcripts, which coincided with previous results in other plant species (Lv et al., 2016; Yan et al., 2020; Hu et al., 2022). We also recognized 363 lncRNAs that exhibit differential expression patterns in response to salt treatments (Supplementary Table S5). Notably, among these DE-lncRNAs, 33, 51, 72, 46, and 26 were exclusively detected at 6 h, 12 h, 24 h, 48 h, and 96 h of salt stress, respectively, when compared to the control (Figure 2A). These findings strongly suggested that the expression of salt-responsive lncRNAs was tightly regulated in a time-dependent manner, consistent with previous reports (Fu et al., 2020; Lee et al., 2022). A prior investigation has indicated that lncRNAs can exert their functions by enhancing gene expression at the transcriptional level (Ørom et al., 2010). Particularly, numerous upregulated DE-lncRNAs were dominant among all salinity conditions, implying a significant impact of high salinity on lncRNA expression.

Plants possess a diverse array of signaling molecules that facilitate the activation of appropriate defense responses in the presence of stress stimuli (Yu et al., 2017). The salt overly sensitive (SOS) signaling pathway, consisting of SOS3, SOS2, and SOS1 from the Na^+^/H^+^ exchanger (NHX), CBL-interacting protein kinase (CIPK), and calcineurin B-like (CBL) gene families, respectively, has been proposed to play a specific role in facilitating cellular signaling and maintaining ion homeostasis in response to salt stress in plants (Ji et al., 2013; Acharya et al., 2023). The CBL-CIPK signaling pathway, in particular, is a crucial component of the SOS pathway. In this work, although we did not directly predict *SOS3*, *SOS2*, and *SOS1* as the lncRNA targets, the identification of salt-inducible DE-lncRNA-targeted CBL-interacting protein kinase 2 (*CIPK2*) and calcineurin B-like protein 1 (*CBL1*) might signify that lncRNAs play a role in regulating the SOS pathway in response to salt stress in radish. Annexin d4 (ANN4), a highly effective endogenous immunomodulatory protein, has been implicated in the cytosolic calcium signaling pathway in response to diverse stressors (Huh et al., 2010; Liao et al., 2017). In our experiment, the expression of TCONS_00115773-regulated *ANN4* was significantly changed under salinity conditions (Table 2; Supplementary Table S7), indicating that lncRNA-*ANN4*-mediated calcium signaling may have a crucial function in enhancing radish resistance to high salinity. Phytohormones extensively participate in the adjustment of plants to unfavorable environmental elicitations. On the other hand, the intricate hormone signaling networks facilitate extensive crosstalk, thereby exerting crucial functions in the mediation of plant defense responses (Verma et al., 2016). Our analysis revealed the identification of DE-target genes involved in hormone signaling and responsive proteins, such as JA-responsive protein 1, and auxin-responsive GH3 family proteins (Tables 1, 2; Supplementary Table S7). Furthermore, we also noted that DE-lncRNA targeted several genes encoding mitogen-activated protein kinase (MAPK) cascades, including MAPK20 and MAPKKK21 (Tables 1, 2; Supplementary Table S7). MAPK cascades are a set of vital signaling kinases that participate in the intracellular transmission of extracellular signals via fine-tuning specific TFs, functional proteins, and transporters (Zhang and Zhang, 2022). Importantly, it is suggested that the interplay between second messengers, hormones, and MAPK modules is instrumental in adapting to diverse environmental cues (Smékalová et al., 2014), suggesting the essential involvement of lncRNAs in sensing and transducing stress signals to activate the defense mechanisms of radish under salt stress conditions.

The interactions between stress-specific TFs and lncRNAs were hypothesized to play a significant role in response to various abiotic stresses, including salt stress (Mirdar Mansuri et al., 2022). For example, in wheat, Shumayla et al. (2017) demonstrated that lncRNAs were co-expressed with the TFs related to WRKY, NAC, MYB, ERF, C3H, C2H2, bZIP, and bHLH families under salt stress conditions; several lncRNAs associated with TFs belonging to ARF, C2C2(Zn), and HSF families were found in duckweed (Fu et al., 2020); in maize, 11 target transcripts of the salt-responsive lncRNAs belonging to seven TF families, including bHLH, C2H2, Hap3/NF-YB, HAS, MYB, WD40, and WRKY, were predicted (Liu et al., 2022a). In this research, a specific group of genes related to TFs were also found in salt-responsive lncRNA–mRNA pairs in radish (Tables 1, 2; Supplementary Table S7). Specifically, the bHLH gene, *bHLH96*, was identified as a cis-regulated target gene of TCONS_00126664; a radish gene homologous to the *Arabidopsis* gene, namely, WRKY transcription factor 40 (*WRKY40*), was predicted to be targeted by TCONS_00007404 and TCONS_00138712. Moreover, TCONS_00146159 was identified to target a TF homologous to Arabidopsis *ERF1A*. The ERF gene *TdERF1* has been found to potentially play a role in the mechanism of salt susceptibility/tolerance by regulating multiple hormone signaling pathways in wheat (Makhloufi et al., 2014), indicating its potential involvement in modulating plant responses to salt constraints. In addition, a considerable number of genes from the TIFY transcription factor family were found to be targeted by DE-lncRNAs. A recent study has reported that TIFYs play an indispensable role in various aspects of plant biology, including growth, development, signal transduction, and response to stress stimuli in plants (Liu et al., 2022b).

A set of evidence also supported the fact that high salinity induces oxidative and osmotic limitations, leading to the accumulation of ROS and dehydration (Cabot et al., 2014; Yang and Guo, 2018; Xiao and Zhou, 2023). Our results indicated that certain targets of DE-lncRNAs related to antioxidants exhibited significant changes in expression levels when subjected to salt conditions (Table 2 and S6). For instance, TCONS_00052720 and TCONS_00031201 were found to fine-tune superoxide dismutase (*SOD*) and peroxidase (*POD*), respectively, which was consistent with the report in cotton (Zhang et al., 2019), suggesting the essential role of lncRNAs in scavenging excess ROS during salt stress. Salt stress also triggers reduced water availability for plants. In this study, several DE-lncRNAs were identified to target transcripts encoding proteins analogous to potato DREB1 with dehydration-responsive element-binding protein 3-like properties (Table 2; Supplementary Table S7), which had been demonstrated to enhance salinity tolerance in transgenic potato plants (Bouaziz et al., 2013).

In addition, several functional proteins and transporters may play considerable roles in adjusting plant responses to salt stress. Chalcone synthase (CHS), a key enzyme in the flavonoid biosynthesis pathway, exerts a significant role in regulating plant growth, development, and defense against abiotic stress (Dao et al., 2011; Hou et al., 2022). The gene encoding chalcone synthase 3 (CHS3) was identified as a trans-acting target gene for four DE-lncRNAs (Table 2; Supplementary Table S7), suggesting that the lncRNA-mediated flavonoid biosynthesis pathway may play an essential role in response to salt stress in radish. Allene oxide cyclases (*AOCs*) were the pivotal genes in the JA biosynthetic pathway involved in regulating plant responses to developmental cues and environmental stresses. Numerous prior studies have demonstrated that the overexpression of allene oxide cyclase (*AOC*) enhances salinity tolerance in plants through the activation of jasmonate signaling (Pi et al., 2009; Zhao et al., 2014). In our study, a considerable number of DEGs encoding allene oxide cyclase 2 (AOC2) and allene oxide cyclase 3 (AOC3) were predicted to be potential targets of DE-lncRNAs (Table 2; Supplementary Table S7). Furthermore, the role of lipoxygenase 3 (LOX3) in *Arabidopsis* has been uncovered as a significant enzyme implicated in JA synthesis in response to salt stress (Ding et al., 2016). Our analysis differentiated that numerous DE-lncRNA-targeted genes encoding LOX3 displayed altered expression levels under salt stress conditions in radish (Table 2; Supplementary Table S7). Together, these findings might imply that the lncRNA-mediated regulation of the JA signaling pathway contributed to radish tolerance to salt stress in a complex and efficient way.

## Data Availability

The datasets presented in this study can be found in online repositories. The names of the repository/repositories and accession number(s) can be found in the article/Supplementary Material.
